# Adaptive Fuzzy Controller Design for Uncertain Robotic Manipulators Subject to Nonlinear Dead Zone Inputs

**DOI:** 10.1155/2022/9173249

**Published:** 2022-09-20

**Authors:** Hua Zhang

**Affiliations:** Zhengzhou Preschool Education College, Zhengzhou 450099, China

## Abstract

This paper comes up with the adaptive fuzzy scheme for multi-degree robotic manipulators with dead zone. Fuzzy logic system is employed to approximate unknown nonlinear functions and input nonlinear functions with dead zones that are introduced. The adaptive fuzzy technique is utilized to deal with the problems of multi-joint robotic manipulators with unknown nonlinear functions in dead zones. Based on Lyapunov criterion, all the states and signals involved in the system are maintained in a certain bounded region and the tracking error converges to a small domain of origin. Finally, a simulation example of robotic manipulators with two joints is structured to confirm the practicability of the raised scheme.

## 1. Introduction

In recent decades, the robotic manipulators are not only the most widely used automated mechanical device in the field of robotics technology but also have an extensive range of applications in daily life. For example, in the medical field, robotic manipulators can easily adapt to the existing operating environments to offer better control for surgeons and ultimately improve surgical outcomes. Besides, the emergence of robotic manipulators can take the place of heavy labor to achieve mechanization and automation of production and operate in harmful environments to ensure personal safety. With the exception of the abovementioned, it can also be applied in industrial manufacturing, medical care, entertainment services, military, semiconductor manufacturing, and so on. Therefore, due to its extensive application and practical value, the research of robotic manipulators is more meaningful.

Since the robotic manipulator is a relatively sophisticated multiple-input-multiple-output (MIMO) nonlinear kinematics system with time-varying, highly coupling, and nonlinear dynamic characteristics, the control of robotic manipulators has been studied extensively and a number of methods have been proposed. In [1], for a redundant manipulator, an adaptive PID fast terminal sliding mode was proposed. A sliding mode and a state observer-based controller were designed to make flexible robotic manipulators perform remote rehabilitation missions in [2]. There are also some other approaches dealing with the robotic manipulators such as sliding mode control [1–6], robust control [3, 7], adaptive fuzzy [8–12], PID controller [13–15]. However, there is a prerequisite for the literature discussed above whose models are all known. To the best of our knowledge, the model equation of the robotic manipulators is uncertain and difficult to establish accurately due to some unavoidable uncertainties such as parameter perturbation, external disturbances, and unmodeled dynamics. Compared with other existing processing methods, adaptive fuzzy control has been widely studied due to its good approximation effect for dealing with a class of unknown and uncertain systems. In [16], the adaptive fuzzy controller was designed to settle multilateral cooperative teleoperation of multiple robotic manipulators under random network-induced delays. A decentralized adaptive fuzzy scheme was proposed for robotic manipulators combining the genetic algorithm and the gradient method in [17]. The adaptive fuzzy control was raised for a robotic manipulator with asymptotic tracking performance in [18]. However, it is noteworthy that the discussion of the aforementioned literature [17, 18] is not involved in the dead zone.

In fact, dead zone is ubiquitous in many industrial systems which affect system operation, especially in the robotic manipulator's system. In [19], a robot manipulator with the dead zone was solved by using the adaptive neural network tracking control method. Backstepping funnel control was used to handle the prescribed performance of robotic manipulators with unknown dead zones in [20]. For flexible manipulators with input dead zones, adaptive neural command filter tracking control was proposed in [21]. However, the models studied in [19–21] were unknown and uncertain, which makes their research methods more consistent with the actual system.

Motivated by the above discussion, the proposed adaptive fuzzy controller for multi-degree robotic manipulators with dead zones will be an interesting and challenging topic for us. In comparison with the available literature, the main contributions of this work are listed as follows:

1. The proposed adaptive fuzzy controller design can be utilized to approximate a large class of uncertain robotic manipulator systems and unknown nonlinear functions under actual conditions. In addition, even though the system model is fully unknown, our controller still works effectively.
2. The uncertain multi-joint robotic manipulator system with dead-zone considered is addressed by the adaptive fuzzy control approach, and the proposed method is valid for other high-order nonlinear systems with dead-zone inputs.

In the end, this paper is settled as below.  Section 2 brings in fuzzy logic system. The models of multi-joint robotic manipulators and input nonlinearity are raised in Section 3.  Section 4 provides a fuzzy controller in the light of the universal approximation criterion for the sake of dealing with robotic manipulators. In Section 5, a simulation case of robotic manipulators with two joints is offered to confirm the feasibility of the proposed method. Besides, conclusions are given to the end.

## 2. Description of the Fuzzy Logic System

Here, we make a short presentation of the fuzzy logic system (FLS). As is known, the FLS is a formidable nonlinear function approximation containing fuzzy IF-THEN rules which are listed as [8].

*R* ^(*K*)^: if *x*_1_ is *P*_1_^*K*^ and … and *x*_*n*_ is *P*_*n*_^*K*^, then $\hat{f}$ is *f*^*K*^, *K*=1, 2,…, *N*,where *N* is fuzzy rules, *P*_1_^*K*^, *P*_2_^*K*^,… and *P*_*n*_^*K*^ are a collection of fuzzy, *f*^*K*^ is the output of the fuzzy singleton in the *K*th rule. In accordance with applying the fuzzifier, product inference, and defuzzifier, the output of the FLS is constructed as [22, 23]

(1) $$\begin{array}{c} \hat{f}\left(\underline{x}\right)=\frac{\sum_{i=1}^{N}f^{i}\left(\prod_{j=1}^{n}\mu_{P_{j}^{i}}\left(x_{j}\right)\right)}{\sum_{i=1}^{N}\left(\prod_{j=1}^{n}\mu_{P_{j}^{i}}\left(x_{j}\right)\right)}\\ =\Theta^{T}R\left(\underline{x}\right)\text{,} \end{array}$$

where *μ*_*P*_*J*_^*i*^_(*x*_*j*_) represents the membership grade which *x*_*j*_ is part of *P*_*j*_^*i*^, Θ^*T*^=[*f*^1^, *f*^2^,…, *f*^*N*^] lies on the following parameters, and *R*^*T*^=[*R*^1^*R*^2^ … *R*^*N*^], where

(2) $$\begin{array}{c} R^{i}\left(\underline{x}\right)=\frac{\prod_{j=1}^{n}\mu_{P_{j}^{i}}\left(x_{j}\right)}{\sum_{i=1}^{N}\left(\prod_{j=1}^{n}\mu_{P_{j}^{i}}\left(x_{j}\right)\right)}. \end{array}$$

Once that fuzzy basis function has been determined, there always exists more than one rule i.e. *∑*_*i*=1_^*n*^(*∏*_*j*=1_^*n*^*μ*_*P*_*j*_^*i*^_(*x*_*j*_)) > 0 [24].

## 3. Dynamic Model of Robotic Manipulators

In this paper, we will consider a more general multi-degree robotic manipulators whose control inputs contain dead zone. The model can be written as

(3) $$\begin{array}{c} M\left(q\right)\ddot{q}+C\left(q,\dot{q}\right)\dot{q}+D\dot{q}+g\left(q\right)=\Psi \left(u\right)\text{,} \end{array}$$

where *q*=[*q*_1_,…,*q*_*n*_]^*T*^ ∈ *R*^*n*^ represents joints position, *u* ∈ *R*^*n*^ is a control signal with dead zones, *M*(*q*) is a inertia matrix, $C\left(q,\dot{q}\right)\dot{q}$ is centrifugal pull, and $D\dot{q}$ and *g*(*q*) are resistance and power of gravity, respectively. *M*(*q*) with respect to *t* total derivative is $\dot{M}$, *D* is a semi-definite symmetric matrix [8, 25].

Let *y*=*q*, $x=\dot{q}$, (2) can be converted into

(4) $$\begin{array}{c} \ddot{y}=M^{-1}\left(y\right)\left[\Psi \left(u\right)-C\left(y,x\right)-D-g\left(y\right)\right]\text{.} \end{array}$$

In the light of (4), the *n* joints robotic manipulators with dead zones can be written as the following compact formula:

(5) $$\begin{array}{c} \ddot{y}=F\left(x\right)+G\left(x\right)\Psi \left(u\right)\text{,} \end{array}$$

where *F*(*x*)=−*M*^−1^(*y*)[*D*+*g*(*y*)+*C*(*y*, *x*)], and *G*(*x*)=*M*^−1^(*y*).

There exists some necessary assumptions that need to be given.

**Assumption 1 .**

Presuming bounded referable signal is *y*_*d*_=[*y*_*d*1_,…,*y*_*dn*_]^*T*^ and *x*=[*x*_1_,…,*x*_*n*_]^*T*^ is known and measurable. Besides, *y*_*d*_ is a known and continuous vector function in a compact set Ω_*y* *d*_.

**Assumption 2 .**

The function *G* is a positive definite matrix, namely, *G*⩾*σ*_0_*I*_*n*×*n*_, with *σ*_0_ ≥ 0.

In accordance with the dynamic model of robotic manipulators system (5), the fuzzy adaptive control approach is designed as follows.

To begin with, the tracking errors are defined by

(6) $$\begin{array}{c} e_{i}=y_{i}-y_{di},i=1,2,\ldots ,n\text{,} \end{array}$$

and the filter tracking error is given as

(7) $$\begin{array}{c} S={\left[S_{1},\ldots ,S_{n}\right]}^{T}\text{,} \end{array}$$

with

(8) $$\begin{array}{c} S_{i}=\left(\begin{array}{c} \lambda_{i},1 \end{array}\right)\left(\begin{array}{c} e_{i}\\ {\dot{e}}_{i} \end{array}\right)\text{.} \end{array}$$

In order to express more clearly, we rewrite (8) into the form of a matrix. Hence, the vector *S* can be expressed as

(9) $$\begin{array}{c} S=C^{T}E\text{,} \end{array}$$

where

(10) $$\begin{array}{c} C^{T}={\left(\begin{array}{ccccc} \lambda_{1} & 1 & 0 & 0\cdots 0 & 0\\ 0 & 0 & \lambda_{2} & 1\cdots 0 & 0\\ \vdots & \vdots & \vdots & \vdots \vdots & \vdots \\ 0 & 0 & 0 & 0\ldots \lambda_{n} & 1 \end{array}\right)}_{\left(n\times 2n\right)}\text{,} \end{array}$$

(11) $$\begin{array}{c} E={\left(\begin{array}{c} e_{1},{\dot{e}}_{1},\ldots ,e_{n},{\dot{e}}_{n} \end{array}\right)}_{\left(2n\times 1\right)}^{T}. \end{array}$$

To proceed given the dynamic of *S*_*i*_ as the form of a matrix, we can also gain

(12) $$\begin{array}{c} {\dot{S}}_{i}=\left(\begin{array}{c} 0,\lambda_{i} \end{array}\right)\left(\begin{array}{c} e_{i}\\ {\dot{e}}_{i} \end{array}\right)+{\ddot{e}}_{i}\text{.} \end{array}$$

Hence, the dynamic of *S* can be given as

(13) $$\begin{array}{c} \dot{S}=C_{r}^{T}E+\ddot{e}\text{,} \end{array}$$

where

(14) $$\begin{array}{c} C_{r}^{T}={\left(\begin{array}{ccccc} 0 & \lambda_{1} & 0 & 0\cdots 0 & 0\\ 0 & 0 & 0 & \lambda_{2}\cdots 0 & 0\\ \vdots & \vdots & \vdots & \vdots \vdots & \vdots \\ 0 & 0 & 0 & 0\ldots 0 & \lambda_{n} \end{array}\right)}_{\left(n\times 2n\right)}\text{,} \end{array}$$

with

(15) $$\begin{array}{c} \ddot{e}=\ddot{y}-{\ddot{y}}_{d}\text{.} \end{array}$$

Then, substituting (15) into (13) yields

(16) $$\begin{array}{c} \dot{S}=C_{r}^{T}E+\ddot{y}-{\ddot{y}}_{d}\text{.} \end{array}$$

### 3.1. Input Nonlinearity

Noting that in the robotic manipulators (3), the dead zone input exists. In this work, this input nonlinearity Ψ_*i*_(*u*_*i*_) can be expressed as [25]

(17) $$\begin{array}{c} \Psi_{i}\left(u_{i}\right)=\left\{\begin{array}{c} Q_{i+}\left(u_{i}\right)\left(u_{i}-u_{iT}\right),\\ 0,\\ Q_{i-}\left(u_{i}\right)\left(u_{i}+u_{it}\right), \end{array}\right.\begin{array}{c} u_{i}>u_{iT}\text{,}\\ -u_{it}⩽u_{i}⩽u_{iT}\text{,}\\ u_{i}<-u_{it}\text{.} \end{array} \end{array}$$

To facilitate the transformation of the dead zone input, here, we suppose that it has some particular properties, i.e., there are two positive constants *M*_*i*+_^*∗*^ and *M*_*i*−_^*∗*^ such that the below conditions can be constructed as [16]

(18) $$\begin{array}{c} \left\{\begin{array}{cc} \left(u_{i}-u_{iT}\right)\Psi_{i}\left(u_{i}\right)\;⩾M_{i+}^{*}{\left(u_{i}-u_{iT}\right)}^{2}\text{,} & u_{i}>u_{iT}\text{,}\\ \left(u_{i}+u_{it}\right)\Psi_{i}\left(u_{i}\right)\;⩾M_{i-}^{*}{\left(u_{i}+u_{it}\right)}^{2}\text{,} & u_{i}<-u_{it}\text{,} \end{array}\right. \end{array}$$

with *β*_*i*_=min {*M*_*i*+_^*∗*^, *M*_*i*−_^*∗*^}.

## 4. The Construction of AFC

In what follows, how to construct a reasonable controller to control the robotic manipulator system (5) is given as follows.

First of all, by substituting (5) into (16), we can obtain

(19) $$\begin{array}{c} \dot{S}=C_{r}^{T}E+F\left(x\right)+G\Psi \left(u\right)-\overset{.}{y_{d}}\text{.} \end{array}$$

Now posing ${\hat{G}}_{1}=G^{-1}$, we have

(20) $$\begin{array}{c} {\hat{G}}_{1}\dot{S}=\hat{G_{1}}\left(C_{r}^{T}E-\overset{.}{y_{d}}+F\left(x\right)\right)+\Psi \left(u\right)\text{.} \end{array}$$

In order to design the optimal controller, (20) is conducted as

(21) $$\begin{array}{c} {\hat{G}}_{1}\dot{S}={\hat{G}}_{1}\left(C_{r}^{T}E-\overset{.}{y_{d}}+F\left(x\right)\right)+\Psi \left(u\right)\\ =\kappa \left(x,m\right)+\Psi \left(u\right)\text{,} \end{array}$$

where $\kappa \left(x,m\right)={\hat{G}}_{1}\left[m+F\left(x\right)\right]$, and $m=C_{r}^{T}E-\overset{.}{y_{d}}$.

**Assumption 3 .**

It is supposed that *κ*_*i*_(*x*, *m*) is a bound continuous function satisfying $\left|\kappa_{i}\left(x,m\right)\right|⩽\beta {\bar{\kappa }}_{i}\left(x\right)$, with *β*=min{*β*_*i*_}.

**Remark 1 .**

The establishment of Assumption 3 is not limited mainly due to the upper limit $\beta {\bar{\kappa }}_{i}\left(x\right)$ is unknown, in addition, *κ*_*i*_(*x*, *m*) is a continuous function in the interval Ω_*x*_ such that $\beta {\bar{\kappa }}_{i}\left(x\right)$ always exists [25].

Here, in light of description of the FLS, ${\bar{\kappa }}_{i}\left(x\right)$ can be depicted as

(22) $$\begin{array}{c} {\hat{\bar{\kappa }}}_{i}\left(x,\Theta \right)=\Theta_{i}^{T}R_{i}\left(x\right)\text{,} \end{array}$$

where *R*_*i*_(*x*) is the FBF which is given by the decision maker and Θ_*i*_ can be tuned based on requirements of the FLS.

We define

(23) $$\begin{array}{c} \Theta_{i}^{*}=arg\;\min_{\Theta_{i}}\;\left[{sup}_{x\in \Omega_{x}}\left|{\bar{\kappa }}_{i}\left(x\right)-{\hat{\bar{\kappa }}}_{i}\left(x,\Theta_{i}\right)\right|\right], \end{array}$$

as the optimal estimate values of Θ_*i*_. It is worth mentioning that artificial constant Θ_*i*_^*∗*^ is introduced only for analysis purposes in the whole process.

Let the estimate error be

(24) $$\begin{array}{c} {\tilde{\Theta }}_{i}=\Theta_{i}-\Theta_{i}^{*}\text{,} \end{array}$$

and the fuzzy approximation error is given as

(25) $$\begin{array}{c} \varepsilon_{i}\left(x\right)={\bar{\kappa }}_{i}\left(x\right)-{\hat{\bar{\kappa }}}_{i}\left(x,\Theta_{i}^{*}\right)\text{,} \end{array}$$

where ${\hat{\bar{\kappa }}}_{i}\left(x,\Theta_{i}^{*}\right)=\Theta_{i}^{*T}R_{i}\left(x\right)$.

It must be emphasized that the input function of FLS must be contained in the scope of Ω_*x*_, otherwise the system cannot operate normally. Assume that *ε*_*i*_(*x*) ∈ Ω_*x*_ is bounded, that is to say, $\left|\varepsilon_{i}\left(x\right)\right|⩽{\bar{\varepsilon }}_{i}$, ${\bar{\varepsilon }}_{i}\in \mathbb{R}$, one can obtain

(26) $$\begin{array}{c} {\hat{\bar{\kappa }}}_{i}\left(x,\Theta_{i}\right)-{\bar{\kappa }}_{i}\left(x\right)={\hat{\bar{\kappa }}}_{i}\left(x,\Theta_{i}\right)-{\hat{\bar{\kappa }}}_{i}\left(x,\Theta_{i}^{*}\right)+{\hat{\bar{\kappa }}}_{i}\left(x,\Theta_{i}^{*}\right)-{\bar{\kappa }}_{i}\left(x\right)\\ ={\hat{\bar{\kappa }}}_{i}\left(x,\Theta_{i}\right)-{\hat{\bar{\kappa }}}_{i}\left(x,\Theta_{i}^{*}\right)-\varepsilon_{i}\left(x\right)\\ ={\tilde{\Theta }}_{i}^{T}R_{i}\left(x\right)-\varepsilon_{i}\left(x\right)\text{.} \end{array}$$

In what follows, let us consider a suitable adaptive fuzzy controller

(27) $$\begin{array}{c} u_{i}=\left\{\begin{array}{c} -Q_{i}\left(t\right)sign\left(S_{i}\right)-u_{it},\\ 0,\\ -Q_{i}\left(t\right)sign\left(S_{i}\right)+u_{iT}, \end{array}\right.\begin{array}{c} S_{i}>0\text{,}\\ S_{i}=0\text{,}\\ S_{i}<0\text{,} \end{array} \end{array}$$

with *Q*_*i*_(*t*)=*z*_0*i*_+*z*_1*i*_|*S*_*i*_|+Θ_*i*_^*T*^*R*_*i*_(*x*), and

(28) $$\begin{array}{c} {\dot{z}}_{0i}=-\zeta_{0i}\sigma_{0i}z_{0i}+\zeta_{0i}\left|S_{i}\right|,z_{0i}\left(0\right)⩾0\text{,}\\ {\dot{\Theta }}_{i}=-\zeta_{1i}\sigma_{1i}\Theta_{i}+\zeta_{1i}\left|S_{i}\right|R_{i}\left(x\right),\Theta_{ij}⩾0\text{,} \end{array}$$

where *ζ*_0*i*_, *ζ*_1*i*_, *σ*_0*i*_, *σ*_1*i*_, *z*_1*i*_ > 0 are marked up to decision makers, *z*_0*i*_ and Θ_*i*_ are estimated values of $z_{0i}^{*}={\bar{\varepsilon }}_{i}$ and Θ_*i*_^*∗*^, respectively.

**Remark 2 .**

With *z*_0*i*_(0)⩾0 and Θ_*i*_(0)⩾0, it is known from that solutions of adaptive laws (28) hold *z*_0*i*_(*t*)⩾0 and Θ_*i*_(*t*)⩾0, for *t* > 0.

Based on Assumption 3 and multiplying both sides of equation (21) by (1/*β*)*S*^*T*^, one can obtain

(29) $$\begin{array}{c} \frac{1}{\beta }S^{T}{\hat{G}}_{1}\left(x\right)\dot{S}=\frac{1}{\beta }S^{T}\kappa \left(x,m\right)+\frac{1}{\beta }S^{T}u\text{,}\\ ⩽\overset{n}{\underset{i=1}{\sum }}\left|S_{i}\right|{\bar{\kappa }}_{i}\left(x\right)+\frac{1}{\beta }S^{T}u\text{.} \end{array}$$

From (26) and (29), one gets

(30) $$\begin{array}{c} \frac{1}{\beta }S^{T}\hat{G_{1}}\left(x\right)\dot{S}⩽\overset{n}{\underset{i=1}{\sum }}\left|S_{i}\right|{\bar{\kappa }}_{i}\left(x\right)+\frac{1}{\beta }S^{T}u\\ ⩽-\overset{n}{\underset{i=1}{\sum }}\left|S_{i}\right|{\tilde{z}}_{0i}-\overset{n}{\underset{i=1}{\sum }}\left|S_{i}\right|{\tilde{\Theta }}_{i}^{T}R_{i}\left(x\right)+\overset{n}{\underset{i=1}{\sum }}\left|S_{i}\right|z_{0i}\\ +\overset{n}{\underset{i=1}{\sum }}\left|S_{i}\right|\Theta_{i}^{T}R_{i}\left(x\right)+\frac{1}{\beta }S^{T}u\text{,} \end{array}$$

with ${\tilde{\Theta }}_{i}=\Theta_{i}-\Theta_{i}^{*}$ and ${\tilde{z}}_{0i}=z_{0i}-z_{0i}^{*}=z_{0i}-{\bar{\varepsilon }}_{i}$.

**Theorem 1 .**

When Assumptions 1–3 are satisfied, for the robotic manipulator system (5) with control law (27) and adaptive laws (28), all the states are remained as a bounded domain and the error function of the closed-loop system tends to be within a small variable range of origin via using adaptive fuzzy approach.

**Proof**

We define

(31) $$\begin{array}{c} V=\frac{1}{2\beta }S^{T}{\hat{G}}_{1}S+\frac{1}{2}\overset{n}{\underset{i=1}{\sum }}\frac{1}{\zeta_{0i}}{\tilde{z}}_{0i}^{2}+\frac{1}{2}\overset{n}{\underset{i=1}{\sum }}\frac{1}{\gamma_{1i}}{\tilde{\Theta }}_{i}^{T}{\tilde{\Theta }}_{i}\text{,} \end{array}$$

(32) $$\begin{array}{c} \dot{V}=\frac{1}{\beta }S^{T}{\hat{G}}_{1}\dot{S}+\overset{n}{\underset{i=1}{\sum }}\frac{1}{\zeta_{0i}}{\tilde{z}}_{0i}{\dot{z}}_{0i}+\overset{n}{\underset{i=1}{\sum }}\frac{1}{\gamma_{1i}}{\tilde{\Theta }}_{i}^{T}{\dot{\Theta }}_{i}\text{,} \end{array}$$

with $\dot{\hat{G_{1}}}=0$. Thus, from (28) and (41), we can obtain the following important inequality. When *S*_*i*_ > 0,

(33) $$\begin{array}{c} \left(u_{i}+u_{it}\right)\Psi_{i}\left(u_{i}\right)=-Q_{i}\left(t\right)sign\left(S_{i}\right)\Psi_{i}\left(u_{i}\right)⩾M_{i-}^{*}Q_{i}^{2}\left(t\right)⩾\beta Q_{i}^{2}\left(t\right)\text{,} \end{array}$$

and when *S*_*i*_ < 0,

(34) $$\begin{array}{c} \left(u_{i}-u_{iT}\right)\Psi_{i}\left(u_{i}\right)=-Q_{i}\left(t\right)sign\left(S_{i}\right)\Psi_{i}\left(u_{i}\right)⩾M_{i+}^{*}Q_{i}^{2}\left(t\right)⩾\beta Q_{i}^{2}\left(t\right)\text{.} \end{array}$$

Hence, we conclude that for all *S*_*i*_ holding

(35) $$\begin{array}{c} -Q_{i}\left(t\right)sign\left(S_{i}\right)\Psi_{i}\left(u_{i}\right)⩾\beta Q^{2}\left(t\right)\text{.} \end{array}$$

Owing to *S*_*i*_^2^ > 0 and *S*_*i*_sign(*S*_*i*_)=|*S*_*i*_|, (35) becomes

(36) $$\begin{array}{c} -Q_{i}\left(t\right)S_{i}^{2}sign\left(S_{i}\right)\Psi_{i}\left(u_{i}\right)⩾\beta Q_{i}^{2}\left(t\right)S_{i}^{2}=\beta Q_{i}^{2}\left(t\right){\left|S_{i}\right|}^{2}\text{.} \end{array}$$

For all *S*_*i*_, we have

(37) $$\begin{array}{c} S_{i}\Psi_{i}\left(u_{i}\right)\leq -\beta Q_{i}\left(t\right)\left|S_{i}\right|\text{.} \end{array}$$

Then, substituting (28), (30), and (37) into (32), we obtain

(38) $$\begin{array}{c} \begin{array}{c} \dot{V}⩽\overset{n}{\underset{i=1}{\sum }}\left|S_{i}\right|z0i+\overset{n}{\underset{i=1}{\sum }}\left|S_{i}\right|\Theta_{i}^{T}R_{i}\left(x\right)+\frac{1}{\beta }\overset{n}{\underset{i=1}{\sum }}S_{i}\Psi_{i}\left(u_{i}\right)-\overset{n}{\underset{i=1}{\sum }}\sigma_{0i}{\tilde{z}}_{0i}z_{0i}\\ -\overset{n}{\underset{i=1}{\sum }}\sigma_{1i}{\tilde{\Theta }}_{i}^{T}\Theta_{i}⩽\overset{n}{\underset{i=1}{\sum }}\left|S_{i}\right|z0i+\overset{n}{\underset{i=1}{\sum }}\left|S_{i}\right|\Theta_{i}^{T}\psi_{i}\left(x\right)+\overset{n}{\underset{i=1}{\sum }}-z_{i}\left(t\right)\left|S_{i}\right|\\ -\overset{n}{\underset{i=1}{\sum }}\sigma_{0i}{\tilde{z}}_{0i}z_{0i}-\overset{n}{\underset{i=1}{\sum }}\sigma_{1i}{\tilde{\Theta }}_{i}^{T}\Theta_{i} \end{array}\\ =-\overset{n}{\underset{i=1}{\sum }}z_{1i}S_{i}^{2}-\overset{n}{\underset{i=1}{\sum }}\sigma_{0i}{\tilde{z}}_{0i}z_{0i}-\overset{n}{\underset{i=1}{\sum }}\sigma_{1i}{\tilde{\Theta }}_{i}^{T}\Theta_{i}\text{.} \end{array}$$

Obviously, we have

(39) $$\begin{array}{c} -\sigma_{0i}{\tilde{z}}_{0i}z_{0i}⩽-\frac{\sigma_{0i}}{2}{\tilde{z}}_{0i}^{2}+\frac{\sigma_{0i}}{2}z_{0i}^{*}2\text{,}\\ -\sigma_{1i}{\tilde{\Theta }}_{i}^{T}\Theta_{i}⩽-\frac{\sigma_{1i}}{2}\left\|{\tilde{\Theta }}_{i}{\|}^{2}+\frac{\sigma_{1i}}{2}\right\|\Theta_{i}^{*}{\|}^{2}\text{.} \end{array}$$

Then, (38) becomes

(40) $$\begin{array}{c} \dot{V}⩽-\overset{n}{\underset{i=1}{\sum }}z_{1i}S_{i}^{2}-\overset{n}{\underset{i=1}{\sum }}\frac{\sigma_{0i}}{2}{\tilde{z}}_{0i}^{2}-\overset{n}{\underset{i=1}{\sum }}-\overset{n}{\underset{i=1}{\sum }}\frac{\sigma_{1i}}{2}{\left\|{\tilde{\Theta }}_{i}\right\|}^{2}\\ +\overset{n}{\underset{i=1}{\sum }}\frac{\sigma_{0i}}{2}z_{0i}^{*}2+\overset{n}{\underset{i=1}{\sum }}\frac{\sigma_{1i}}{2}{\left\|\Theta_{i}^{*}\right\|}^{2} \end{array}$$

Since ${\hat{G}}_{1}⩾\sigma_{g0}I_{n}$, then we have

(41) $$\begin{array}{c} S^{T}G^{-1}S=S^{T}{\hat{G}}_{1}S⩽\frac{1}{\sigma_{g0}}{\left\|S\right\|}^{2}\text{.} \end{array}$$

From (40) and (41), one has

(42) $$\begin{array}{c} \dot{V}⩽-\mu V+\tau \text{,} \end{array}$$

where

(43) $$\begin{array}{c} \tau =\overset{n}{\underset{i=1}{\sum }}\frac{\sigma_{0i}}{2}z_{0i}^{*}2+\overset{n}{\underset{i=1}{\sum }}\frac{\sigma_{1i}}{2}{\left\|\Theta_{i}^{*}\right\|}^{2}\text{,}\\ \omega =\min\;\left\{\min_{i}\;\left\{2\eta \sigma_{g0}z_{1i}\right\},\min_{i}\;\left\{\zeta_{0i}\sigma_{0i}\right\},\min_{i}\;\left\{\zeta_{1i}\sigma_{1i}\right\}\right\}\text{.} \end{array}$$

Multiplying (42) by *e*^*ωt*^ yields

(44) $$\begin{array}{c} \frac{d}{dt}\left(Ve^{\omega t}\right)⩽\tau e^{\omega t}\text{.} \end{array}$$

Then, integrating over the interval [0, t] of the (44), we can get

(45) $$\begin{array}{c} 0⩽V\left(t\right)⩽\frac{\tau }{\omega }+\left(V\left(0\right)-\frac{\tau }{\omega }\right)e^{-\omega t}\text{.} \end{array}$$

By utilizing (31), *V*(0) is built as

(46) $$\begin{array}{c} V\left(0\right)=\frac{1}{2\beta }S{\left(0\right)}^{T}\hat{G_{1}}S0+\frac{1}{2}\overset{n}{\underset{i=1}{\sum }}\frac{1}{\zeta_{0i}}z_{0i}0-z_{0i}^{*}{\;}^{2}\\ +\frac{1}{2}\overset{n}{\underset{i=1}{\sum }}\frac{1}{\zeta_{1i}}\Theta_{i}0-\Theta_{i}^{*}{\;}^{T}\Theta_{i}0-\Theta_{i}^{*}\text{,} \end{array}$$

where $\hat{G_{1}}⩾\sigma_{g1}I_{n}$, *σ*_*g*1_ > 0.

In the light of (31) and (45), one can achieve

(47) $$\begin{array}{c} \left|S_{i}\right|⩽{\left(\frac{2\beta }{\sigma_{g1}}\left(\frac{\tau }{\omega }+\left(V\left(0\right)-\frac{\tau }{\omega }\right)e^{-\omega t}\right)\right)}^{1/2}\text{.} \end{array}$$

From the above inequality, it can be concluded that the exponential solution of *S*_*i*_ gradually tends to be bounded, where Ω_*S*_*i*__={*S*_*i*_||*S*_*i*_| ⩽ (2*η*/*σ*_*g*1_*τ*/*ω*)^1/2^}, and the proof of the theorem is finished.

**Remark 3 .**

**Figure 1:**
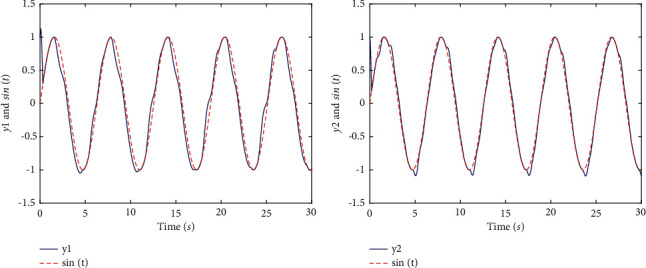
The tracking performance of two-link robotic manipulators systems without dead zones (with *α*=1). (a) Tracking of *y*_1_ and sin *t*. (b) Tracking of *y*_2_ and sin *t*.

**Figure 2:**
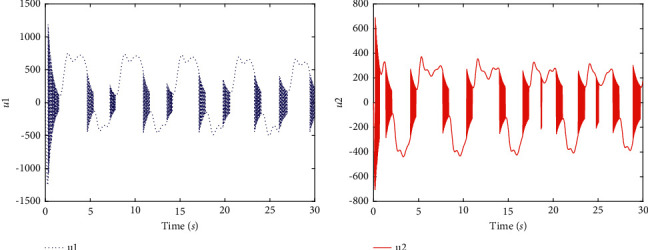
Simulation results (with *α*=1). (a) The controller *u*_1_. (b) The controller *u*_2_.

It is worth mentioning that there is a special example Ψ_*i*_(*u*_*i*_)=*u*_*i*_, that is, when the system model does not contain nonlinear input functions with dead zones, the controller we structured is still applicable to system (5), and the tracking performance are shown in Figures 1 and 2.

**Remark 4 .**

(1) If *u*_*iT*_=*u*_*it*_=*u*_*ii*_ are established, (27) can be transformed into
  (48) $$\begin{array}{c} u_{i}=-\left(u_{ii}+Q_{i}\left(t\right)\right)sign\left(S_{i}\right)\text{,} \end{array}$$
where *Q*_*i*_(*t*)=*z*_0*i*_+*z*_1*i*_|*S*_*i*_|+Θ^*T*^*R*_*i*_(*x*).
(2) It is worth highlighting that the approximation effect of the robotic manipulator is significantly better when the function sign(.) is substituted for a smooth function arcsin(.), arctan(.), and so on.

## 5. Simulation Results

With the aim of checking the control efficiency of the proposed scheme, let us consider robotic manipulators with two single joints without disturbance.

(49) $$\begin{array}{c} \left(\begin{array}{c} {\ddot{q}}_{1}\\ {\ddot{q}}_{2} \end{array}\right)={\left(\begin{array}{cc} M_{11} & M_{12}\\ M_{21} & M_{22} \end{array}\right)}^{-1}\left\{\left(\begin{array}{c} \Psi_{1}\left(u_{1}\right)\\ \Psi_{2}\left(u_{2}\right) \end{array}\right)-\left(\begin{array}{cc} C_{11} & C_{12}\\ C_{21} & C_{22} \end{array}\right)\left(\begin{array}{c} {\dot{q}}_{1}\\ {\dot{q}}_{2} \end{array}\right)-\left(\begin{array}{c} g_{11}\\ g_{21} \end{array}\right)\right\}, \end{array}$$

where *M*_11_=*b*_1_+4*b*_4_cos*q*_2_, *M*_12_=*b*_2_+*b*_4_cos*q*_2_, *M*_21_=*b*_1_+*b*_4_cos*q*_2_, *M*_22_=*b*_3_, $C_{11}=-b_{4}{\dot{q}}_{2}\sin q_{2}$, $C_{12}=-b_{4}\sin q_{2}\left({\dot{q}}_{1}+{\dot{q}}_{2}\right)$, $C_{21}=b_{4}{\dot{q}}_{1}\sin q_{2}$, *C*_22_=0, *g*_11_=*c*_1_cos*q*_1_+*c*_2_cos (*q*_1_+*q*_2_), *g*_21_=*c*_2_cos (*q*_1_+*q*_2_) and the parameters are designed below without disturbance: *b*_1_=200.03, *b*_2_=23.7, *b*_3_=122.8, *b*_4_=25.3, *c*_1_=784.9, *c*_2_=245.35.

Let us denote *y*=[*y*_1_, *y*_2_]^*T*^=[*q*_1_, *q*_2_]^*T*^, $x={\left[x_{1},x_{2},x_{3},x_{4}\right]}^{T}={\left[q_{1},q_{2},{\dot{q}}_{1},{\dot{q}}_{2}\right]}^{T}$, and Ψ(*u*)=[Ψ_1_(*u*_1_), Ψ_2_(*u*_2_)], thus, (49) is depicted as

(50) $$\begin{array}{c} \ddot{y}=F\left(x\right)+G\left(x\right)\Psi \left(u\right)\text{.} \end{array}$$

The input nonlinearities Ψ_1_(*u*_1_) and Ψ_2_(*u*_2_) are arranged as

(51) $$\begin{array}{c} \Psi_{1}\left(u_{1}\right)=\left\{\begin{array}{cc} \left(u_{1}-2\right)\left(1-0.3\;\sin u_{1}\right)\text{,} & u_{1}>2\text{,}\\ 0\text{,} & -2⩽u_{1}⩽2\text{,}\\ \left(u_{1}+2\right)\left(0.8-0.3\;\cos u_{1}\right)\text{,} & u_{1}<-2\text{,} \end{array}\right. \end{array}$$

and Ψ_2_(*u*_2_) is supposed to be

(52) $$\begin{array}{c} \Psi_{2}\left(u_{2}\right)=\left\{\begin{array}{c} \left(u_{2}-5\right)\left(1-0.3\;\sin u_{2}\right),u_{2}>5\text{,}\\ 0,-5\leq u_{2}\leq 5\text{,}\\ \left(u_{2}+5\right)\left(0.8-0.3\;\cos u_{2}\right),u_{2}<-5\text{.} \end{array}\right. \end{array}$$

In the simulation process, a fuzzy logic system is used, which contains four identical Gaussian functions. Each Gaussian function is distributed in the interval [−10,10] and their standard deviation is selected as 0.9. The input vector of the fuzzy system $\Theta^{T}R\left(\underline{x}\right)$ is *x*=[*x*_1_, *x*_2_, *x*_3_, *x*_4_]^*T*^. Here, we set the step size as 5 so that there exists 5 × 5 × 5 × 5=625 rules in the FLS. The parameters in the simulation process are designed as *ζ*_01_=31, *ζ*_02_=79, *ζ*_11_=*ζ*_12_=4000, *σ*_01_=*σ*_02_=0.001, *σ*_11_=*σ*_12_=0.0005, *λ*_1_=*λ*_2_=2, and *z*_11_=*z*_12_=2. Simulation initial parameters of adaptive laws and control law are chosen as *z*_01_=*z*_02_=0, Θ_1*j*_=Θ_2*j*_, *j*=1,…, 625, and *u*_1*T*_=*u*_2_*T*=2, *u*_1_*t*=*u*_2_*t*=5. Original values of the robotic manipulator system are designed as *x*(0)=[1,2,3,4]^*T*^ and *y*(0)=[1,1]^*T*^. Here, one can see that sign(*S*_*i*_) is a piecewise discontinuous function that can take the place of tanh (*z*_*si*_*S*_*i*_) with *z*_*si*_=20, *i*=1, 2. It is noteworthy that our target is to make the robotic manipulator system joints *q*_1_ and *q*_2_ to track the desired given trajectories *y*_*d*1_=*y*_*d*2_=sin *t*.

**Figure 3:**
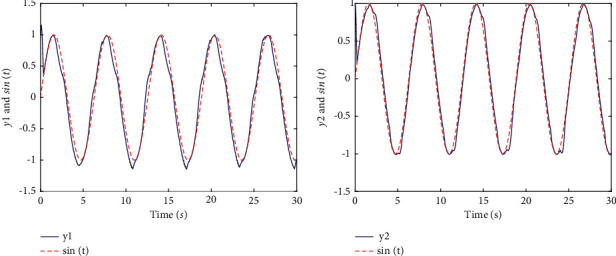
The tracking performance of two-link robotic manipulators systems with dead zones (with *α*=1). (a) Tracking of *y*_1_ and sin *t*. (b) Tracking of *y*_2_ and sin *t*.

**Figure 4:**
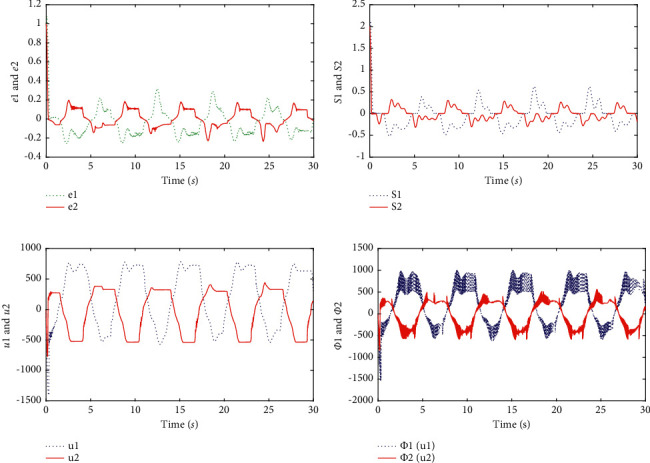
Simulation results (with *α*=1). (a) The tracking error *e*_1_ and *e*_2_. (b) The sliding mode *S*_1_ and *S*_2_. (c) The controller *u*_1_ and *u*_2_. (d) Nonlinear functions Ψ_1_(*u*_1_) and Ψ_2_(*u*_2_).

Finally, it is apparent that presents the good tracking performances of the two-link robotic manipulators with dead zones in Figure 3.  Figure 4 shows transient behaviors of the tracking error, sliding mode, controllers, and nonlinear functions with dead zones. In terms of Remark 4, it is noticeable that the tracking effect in Figure 1 is the same as Figure 3 and the range of variation of controllers with different parameter values are shown in Figure 2.

## 6. Conclusion

This paper adopts the adaptive fuzzy control approach for uncertain multi-degree robotic manipulators with a dead zone. The fuzzy logic system has been presented to dispose of unknown and uncertain nonlinear functions of the robotic manipulator. It has been proven in accordance with Lyapunov criterion that the proposed adaptive fuzzy control scheme can guarantee that the states and signals of the whole closed-loop system tend toward bounded and the tracking errors are close to small regions of origin gradually. The practicability of the proposed approach is affirmed by an example of robotic manipulators with two joint. The deficiency of this paper is that the tracking error observed from the image is relatively large, how to reduce the tracking error will be our future research direction.

## Data Availability

All datasets generated for this study are available from the corresponding author upon request.
